# Author Correction: Emergent patterns of patchiness differ between physical and planktonic properties in the ocean

**DOI:** 10.1038/s41467-026-71994-9

**Published:** 2026-04-20

**Authors:** Patrick Clifton Gray, Emmanuel Boss, Guillaume Bourdin, Mission Microbiomes AtlantECO, A. Bourdais, C. Bowler, C. Moulin, C. de Vargas, D. Iudicone, D. Couet, E. Catafort, E. Boss, E. Petit, E. Mayeux, F. Lombard, J. Schramm, L. Guidi, M. Moll, P. Wincker, R. Laxenaire, R. Troublé, S. Sanchez, S. Pesant, T. Linkowski, Tara Pacific Consortium, C. Bowler, C. Moulin, C. de Vargas, E. Boss, R. Troublé, S. Planes, D. Allemand, N. Djerbi, B. C. C. Hume, T. Röthig, M. Ziegler, L. Paoli, J. M. Flores, N. Lang-Yona, P. Conan, P. E. Galand, E. Douville, S. Agostini, Y. Kitano, O. da Silva, D. R. Cronin, E. Armstrong, J. -M Aury, B. Banaig, Barbe, C. Belser, E. Beraud, E. Boissin, G. Klinges, E. Bonnival, G. Bourdin, E. Bourgois, Q. Carradec, S. Pesant, M. Miguel-Gordo, N. Cassar, S. G. John, N. R. Cohen, G. Reverdin, J. Filée, J. R. Dolan, G. Dominguez Herta, J. Du, D. Forcioli, R. Friedrich, P. Furla, J. -F Ghiglione, E. Gilson, G. Gorsky, M. Guinther, N. Haëntjens, N. Henry, M. Hertau, C. Hochart, G. Iwankow, L. Karp-Boss, R. L. Kelly, I. Koren, K. Labadie, J. Lancelot, J. Lê-Hoang, R. Lemee, Y. Lin, F. Lombard, D. Marie, R. McMind, M. Trainic, D. Monmarche, Y. Mucherie, B. Noel, A. Ottaviani, M. -L Pedrotti, C. Pogoreutz, J. Poulain, M. Pujo-Pay, S. Reynaud, S. Romac, E. Rottinger, A. Rouan, H. -J Ruscheweyh, G. Salazar, M. B. Sullivan, S. Sunagawa, O. P. Thomas, A. Vardi, R. Vega-Thunder, C. R. Voolstra, P. Wincker, A. Zahed, T. Zamoum, D. Zoccola, Mission Microbiomes CEODOS Chile, Camila Fernández, Alejandro Maass, Chris Bowler, Leonardo Castro, Giovanni Daneri, Colomban De Vargas, Damien Eveillard, Laura Farías, Humberto González, Lionel Guidi, Fabien Lombard, Daniele Iudicone, Silvio Pantoja, Renato Quiñones, Romain Trouble, Patrick Wincker, Yoav Lehahn

**Affiliations:** 1 School of Marine Sciences, University of Maine, Orono, ME USA; 2 Department of Marine Geosciences, Charney School of Marine Sciences, University of Haifa, Haifa, Israel; 3 Tara Ocean Foundation, Paris, France; 4 Centre National de la Recherche Scientifique, Paris, France; 5 Sorbonne Université, Paris, France; 6 Stazione Zoologica Anton Dohrn, Naples, Italy; 7 World Courier, Paris, France; 8 Centre de l’énergie atomique, Paris, France; 9 EMS Sistemas, Barcelona, Spain; 10 Ecole Normale Supérieure, Paris, France; 11 European Molecular Biology Laboratory, Heidelberg, Germany; 12 PSL Research University: EPHE-UPVD-CNRS, USR 3278 CRIOBE, Université de Perpignan, Perpignan, France; 13 Centre Scientifique de Monaco, Principality of Monaco, Monaco, Monaco; 14 Université Côte d’Azur, CNRS, Inserm–IRCAN, Nice, France; 15 Department of Biology, University of Konstanz, Konstanz, Germany; 16 Aquatic Research Facility, Environmental Sustainability Research Centre, University of Derby, Derby, United Kingdom; 17 Department of Animal Ecology & Systematics, Justus Liebig University, Giessen, Germany; 18 Department of Biology, Institute of Microbiology and Swiss Institute of Bioinformatics, ETH Zürich, Zürich, Switzerland; 19 Weizmann Institute of Science, Dept. Earth and Planetary Science, Rehovot, Israel; 20 Weizmann Institute of Science, Dept. Plant and Environmental Science, Rehovot, Israel; 21 Sorbonne Université, CNRS, LOMIC, Observatoire Océanologique de Banyuls, Banyuls-sur-Mer, France; 22 Sorbonne Université, CNRS, LECOB, Observatoire Océanologique de Banyuls, Banyuls-sur-Mer, France; 23 Laboratoire des Sciences du Climat et de l’Environnement, LSCE/IPSL, CEA-CNRS-UVSQ, Université Paris-Saclay, Gif-sur-Yvette, France; 24 Shimoda Marine Research Center, University of Tsukuba, Shizuoka, Japan; 25 National Institute of Environmental Science, Tsukuba, Japan; 26 Sorbonne Université, Institut de la Mer de Villefranche sur mer, Laboratoire d’Océanographie de Villefranche, Villefranche-sur-Mer, France; 27 The Ohio State University, Departments of Microbiology and Civil, Environmental and Geodetic Engineering, Columbus, Ohio, United States of America and The Ohio State University, Departments, Columbus, Ohio, United States of America; 28 Génomique Métabolique, Genoscope, Institut François Jacob, CEA, CNRS, Univ Evry, Université Paris-Saclay, Evry, France; 29 Oregon State University, Department of Microbiology, Corvallis, Oregon, United States of America; 30 Sorbonne Université, CNRS, Station Biologique de Roscoff, AD2M, UMR 7144, ECOMAP, Roscoff, France; 31 PANGAEA, Data Publisher for Earth and Environment Science, Bremen, Germany & MARUM—Center for Marine Environmental Sciences, Universität Bremen, Bremen, Germany; 32 Marine Biodiscovery Laboratory, School of Chemistry and Ryan Institute, National University of, Ireland Galway, Ireland; 33 Division of Earth and Ocean Sciences, Duke University, Durham, North Carolina United States of America; 34 Laboratoire des Sciences de l’Environnement Marin LEMAR., UMR 6539 UBO/CNRS/IRD/IFREMER, Institut Universitaire Européen de la Mer IUEM, Brest, France; 35 Department of Earth Sciences, University of Southern California, Los Angeles, California, United States of America; 36 Marine Chemistry and Geochemistry Department, Woods Hole Oceanographic Institution, Falmouth, Massachussettes, United States of America; 37 Institut Pierre Simon Laplace, CNRS/IRD/MNHN LOCEAN. Sorbonne-Université Paris, Paris, France; 38 Laboratoire Evolution, Génomes, Comportement et Ecologie, CNRS/Université Paris-Saclay, Avenue de la Terrasse, Gif sur Yvette, France; 39 Center for Oceanographic Research in the Eastern South Pacific COPAS COASTAL, Concepción, Chile; 40 Department of Oceanography, University of Concepción, Concepción, Chile; 41 Laboratory of Microbial Oceanography, UMR7621, Banyuls sur Mer CNRS, Banyuls-sur-Mer, France; 42 Department of Mathematical Engineering, University of Chile, Santiago, Chile; 43 Center for Mathematical Modeling, University of Chile and IRL2807 CNRS, Santiago, Chile; 44 Millennium Institute Center for Genome Regulation, Santiago, Chile; 45 Research Federation for the Study of Global Ocean Systems Ecology and Evolution, FR2022/Tara Oceans GOSEE, Paris, France; 46 Centro de Investigación en Ecosistemas de la Patagonia (CIEP), Coyhaique, Chile; 47 Nantes Université, École Centrale Nantes, CNRS, LS2N, UMR 6004, Nantes, France; 48 Instituto Milenio en Socio-Ecología Costera (SECOS), Santiago, Chile; 49 Center for Climate and Resilience Research (CR2), University of Chile, Santiago, Chile; 50 Centro FONDAP de Investigación en Dinámica de Ecosistemas Marinos de Altas Latitudes (IDEAL), Punta Arenas, Chile; 51 Instituto de Ciencias Marinas y Limnológicas, Universidad Austral de Chile, Valdivia, Chile; 52 Interdisciplinary Center for Aquaculture Research (INCAR), Universidad de Concepción, Concepción, Chile

**Keywords:** Ocean sciences, Ecology

Correction to: *Nature Communications* 10.1038/s41467-025-56794-x, published online 20 February 2025

In the version of the article initially published, the Mission Microbiomes CEODOS Chile was missing from the author list and has now been added. Additionally, the surname of D. Iudicone (Mission Microbiomes AtlantECO) appeared incorrectly (as Ludicone) and has now been corrected. These corrections have been made to the HTML and PDF versions of the article.

