# Supplementary material for: Emergent patterns of patchiness differ between physical and planktonic properties in the ocean
Source: Nat Commun. 2025 Feb 20;16:1808. doi: 10.1038/s41467-025-56794-x (PMC11842825; doi:10.1038/s41467-025-56794-x)
Supplement: Supplementary file 1 — Supplementary Information [file 41467_2025_56794_MOESM1_ESM.pdf]

Supplemental material for the manuscript:

Emergent patterns of patchiness differ between physical and planktonic properties in the ocean

Patrick Clifton Gray*,[1,2], Emmanuel Boss[1], Guillaume Bourdin[1], Mission Microbiomes AtlantECO[‡], Tara Pacific Consortium[‡], Yoav Lehahn*[2]

1. School of Marine Sciences, University of Maine, Orono, ME, USA
2. Department of Marine Geosciences, Charney School of Marine Sciences, University of Haifa, Haifa, Israel

[‡] Members of the Mission Microbiomes AtlantECO and the Tara Pacific Consortium are listed in the Acknowledgments.

*Corresponding authors: Patrick Gray (patrick.gray@maine.edu) and Yoav Lehahn (ylehahn@univ.haifa.ac.il)

Supplemental Methods

All MODIS Aqua SST and chlorophyll-a imagery was downloaded for August 2016. This period was selected both to overlap with the R/V Tara data and to conduct the analysis on data well before the known degradation of the MODIS-Aqua ocean color products (10.1117/12.2676873). SST and chl-a data were conservatively filtered using only data where both the chl-a and SST product flags indicated no warnings of bad or suspicious data. The variance slope calculation was done on each individual image to preserve spatial patterns and these individual calculations of variance slope were then mean binned into 2° x 2° pixels.

Supplemental Figures

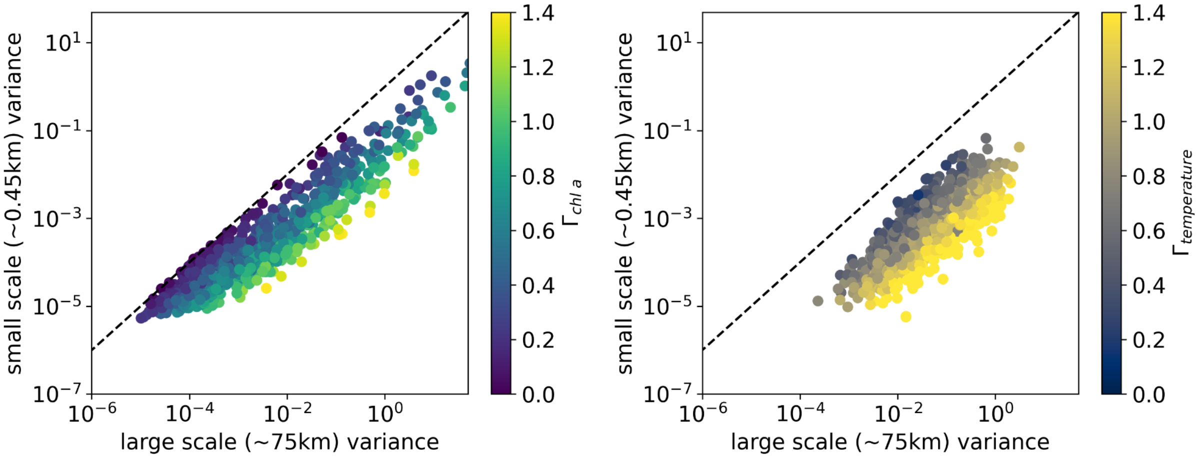

Figure S1. Small scale (1km) vs large scale (75km) variance colored by the variance slope (Γ) for chlorophyll-a and temperature. This illustrates that the Γ value is qualitatively a change in the ratio small to large scale variance where more large-scale variance increases the value of Γ and more small-scale variance decreases it.

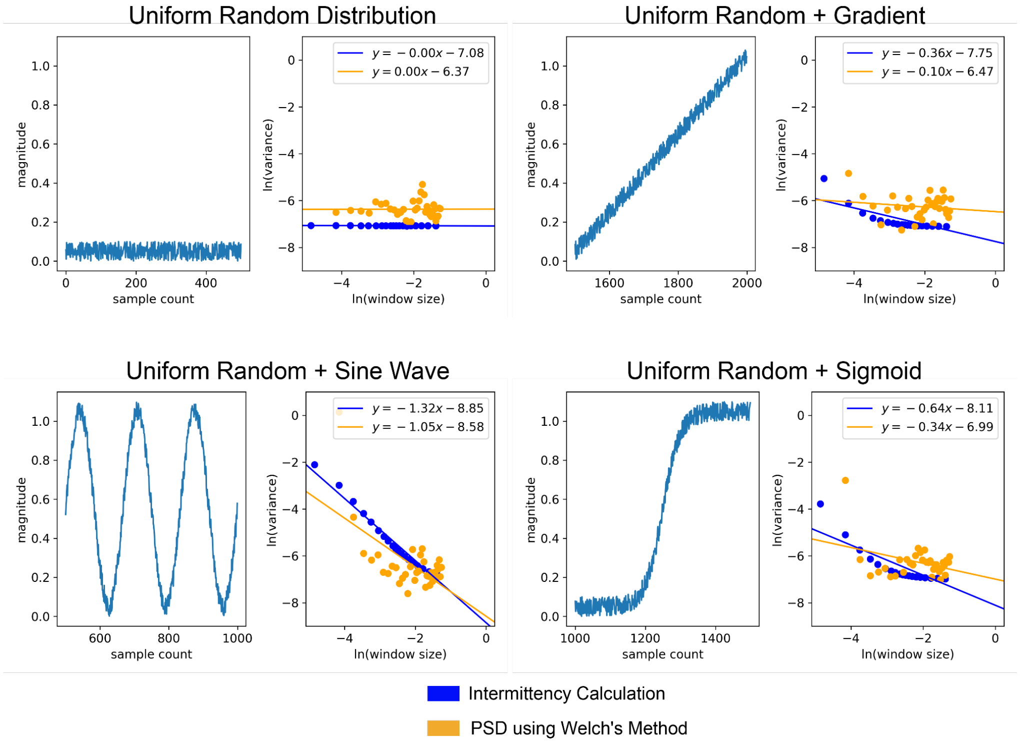

Figure S2. Comparisons of variance slope (Γ) and a typical power spectral density slope calculated via Welch's Method for four different synthetic patterns. This shows both the similarity to power spectra calculations and how much less sensitive this method is to noise.

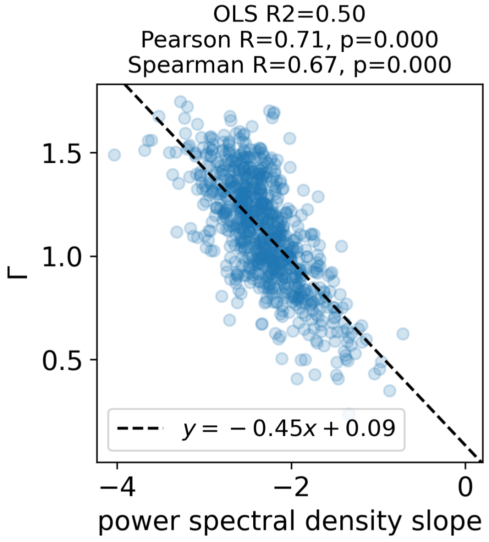

Figure S3. The relationship of variance slope (Γ) and the power spectral density slope for a large dataset of temperature collected on the R/V *Tara* showing the tight correspondence and the formula for approximate conversion between the two.

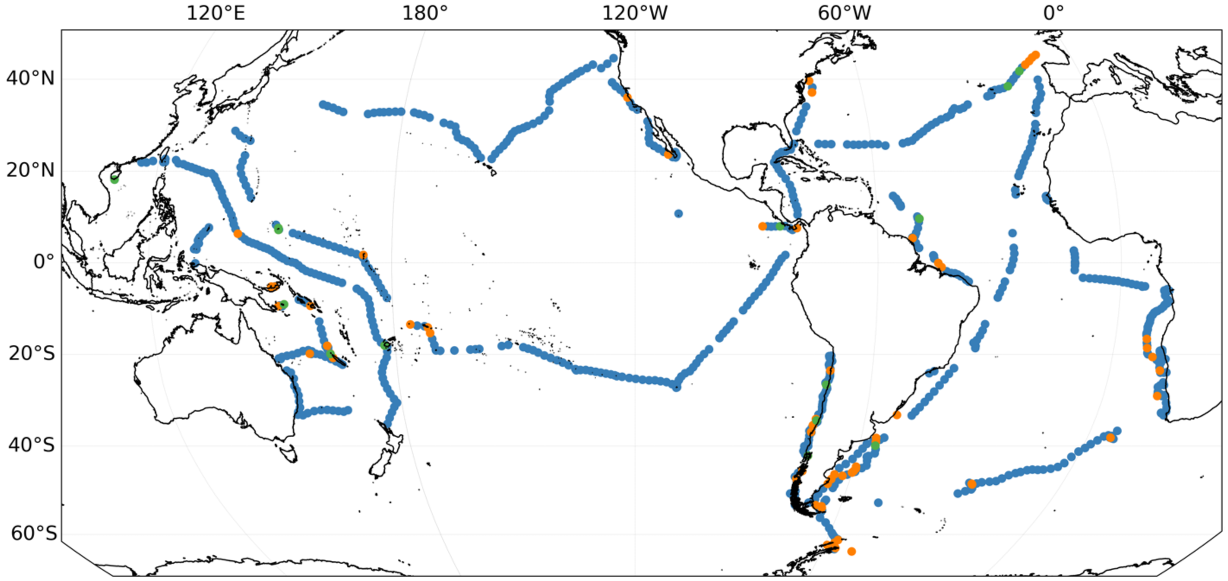

Figure S4. Here we highlight regions where chlorophyll-a is patchier than temperature (blue), where temperature is patchier than chlorophyll-a (orange), and where the variance slope (Γ) values are within 0.02 of each other (green).

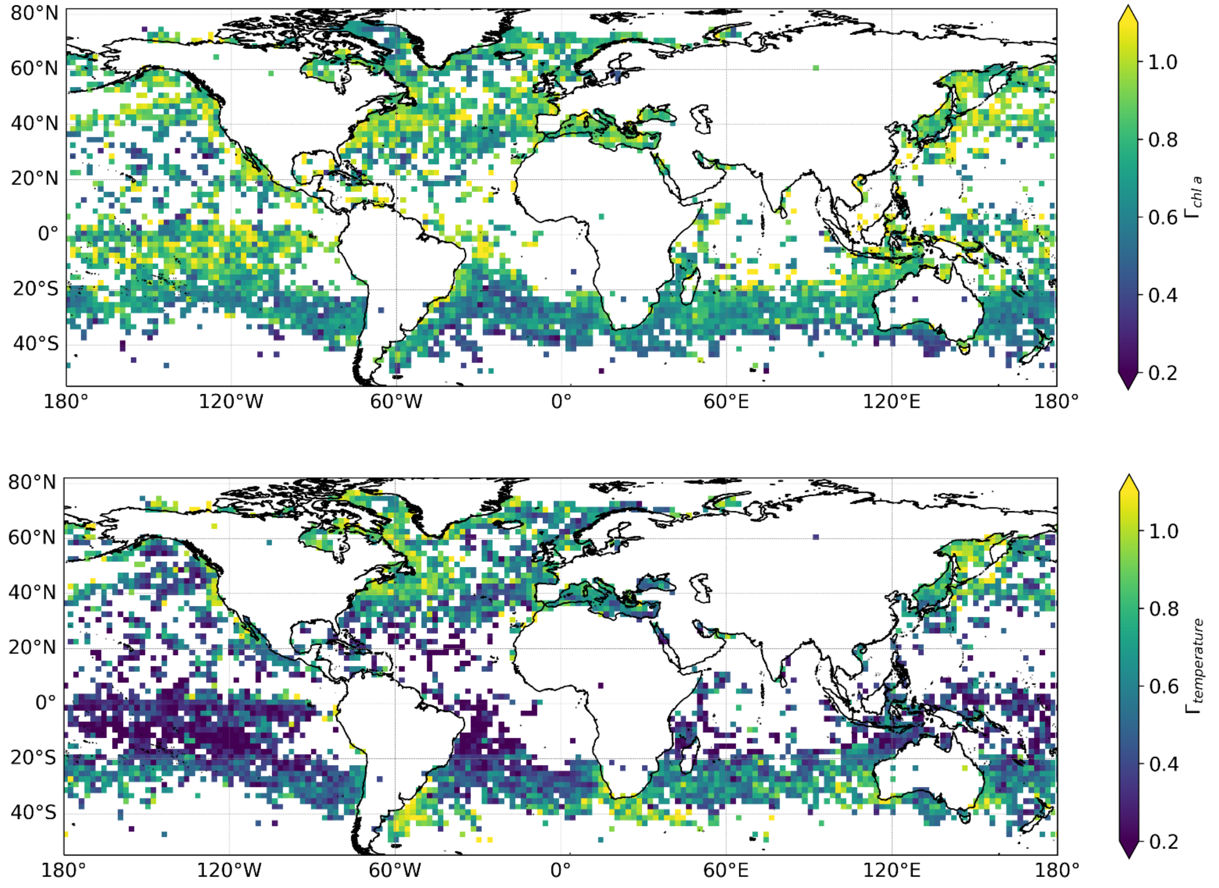

Figure S5. Variance slope (Γ) for chlorophyll-a (chla) and temperature from MODIS data. Note that the calculation here is done from 3 km to 100 km (dx=1) and then interpolated to a 2x2° grid where all slope values within each grid square are averaged. This was to match as closely as possible the R/V *Tara* analysis which goes from .6 km to 100 km (dx=0.2km).

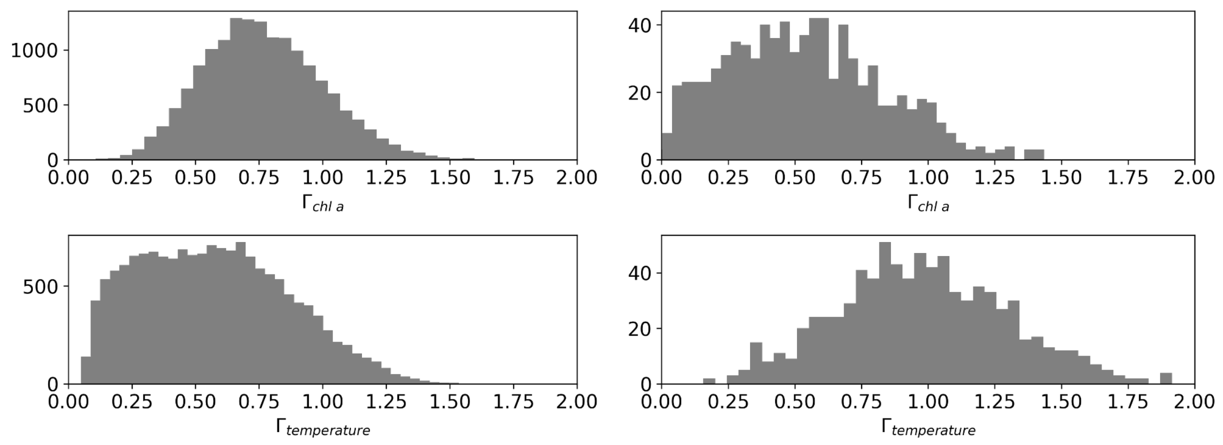

Figure S6. Satellite and R/V *Tara* chlorophyll-a (chl a) and temperature variance slopes (Γ). The satellite data is from MODIS Aqua and consists of data from all of Aug 2016. This shows the mismatch between in situ (right) and satellite (left) derived Γ values where satellite Γ_{chla} is higher than satellite $\Gamma_{temperature}$, exactly the opposite of what is observed in situ. Note that the in situ histograms are slightly different than those in Figure 3 because the histograms shown here have been calculated to match the resolution of MODIS from 3km to 100km.

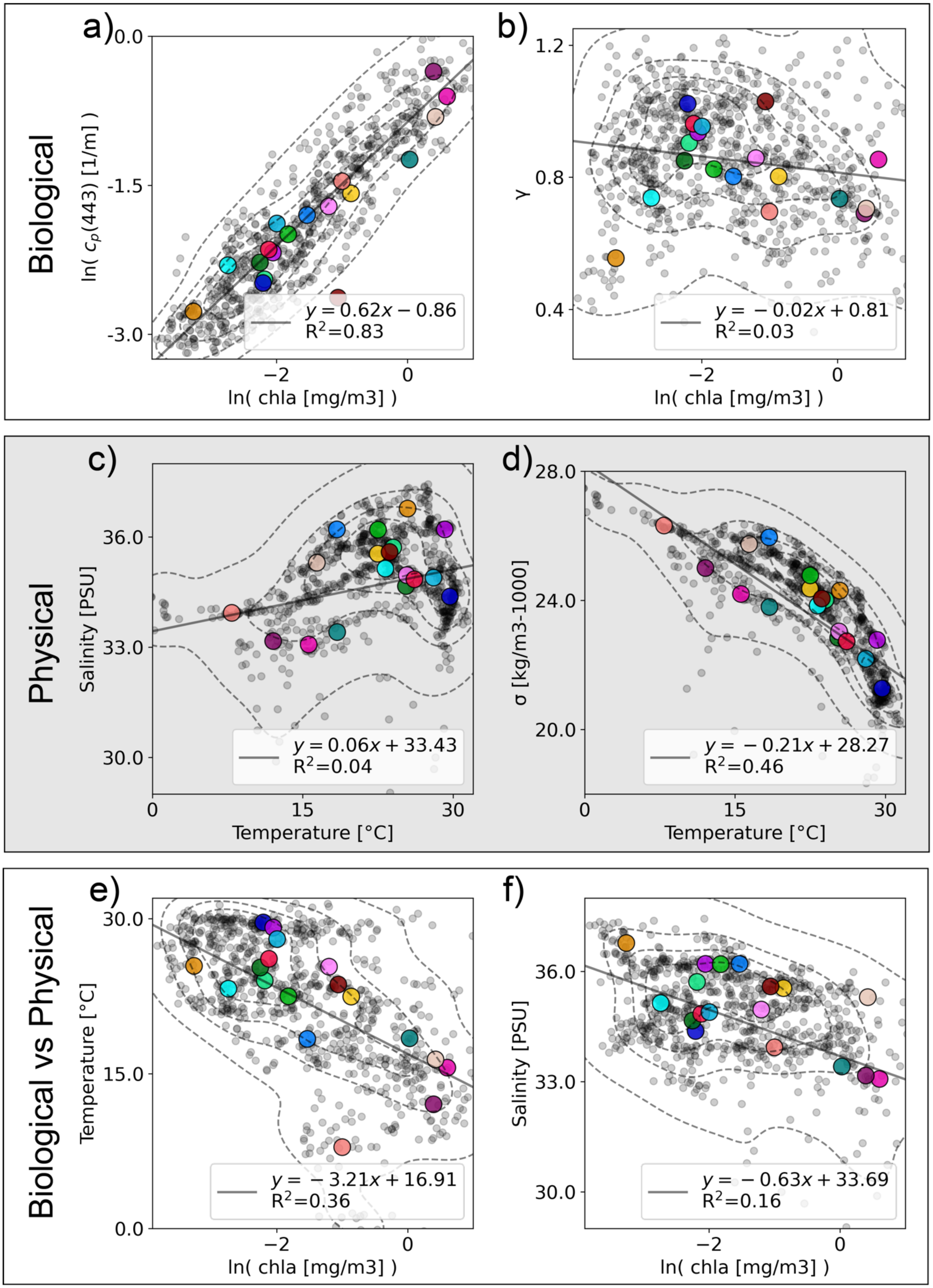

Biological
a)
ln(c_p(443) [1/m])
ln(chla [mg/m3])
$y = 0.62x - 0.86$
R^2=0.83
b)
γ
ln(chla [mg/m3])
$y = -0.02x + 0.81$
R^2=0.03
Physical
c)
Salinity [PSU]
Temperature [°C]
$y = 0.06x + 33.43$
R^2=0.04
d)
σ [kg/m3-1000]
Temperature [°C]
$y = -0.21x + 28.27$
R^2=0.46
Biological vs Physical
e)
Temperature [°C]
ln(chla [mg/m3])
$y = -3.21x + 16.91$
R^2=0.36
f)
Salinity [PSU]
ln(chla [mg/m3])
$y = -0.63x + 33.69$
R^2=0.16

Figure S7. Relationships between the absolute values of the variables across the dataset, rather than their patchiness. Larger colored markers correspond with the Longhurst provinces from Figure 5. Contours partition the data's probability mass function into five equal levels. N.b. correlations are shown for all data, not the Longhurst province means, and all plots have a p-value < 0.001. Here we emphasize that despite the strong relationships between the values of some parameters in this figure their patchiness is not necessarily correlated and that some with no relationship in absolute value have fairly strong relationships in patchiness (Figure 4).

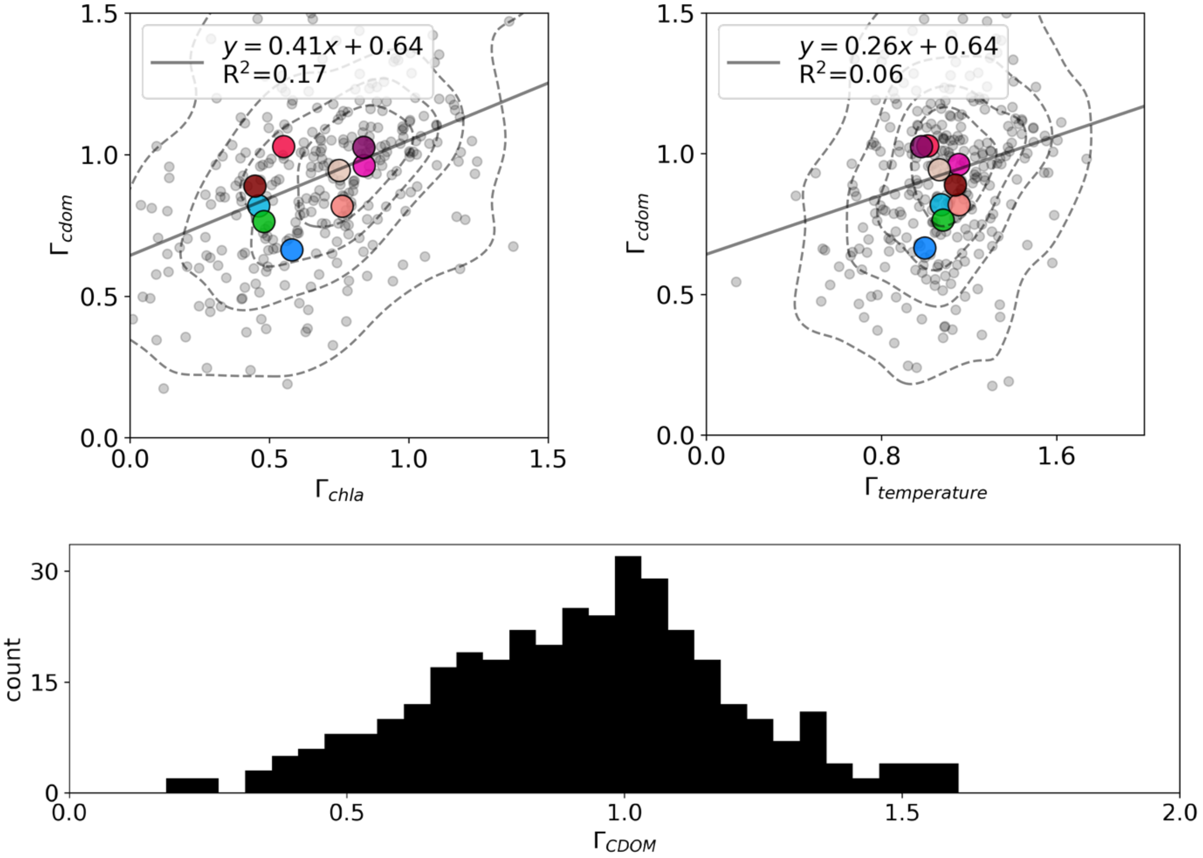

Figure S8. Relationships and distribution of colored dissolved organic matter (CDOM) and CDOM variance slope (Γ). Top left panel shows Γ_{chla} vs Γ_{cdom}. Top right shows

$\Gamma_{\text{temperature}}$ vs Γ_{cdom}. Bottom panel shows the distribution of Γ_{cdom} over the dataset. These relationships are intermediate between typical biological parameters (chla and particulate attenuation) and physical parameters with somewhat more variance explained by temperature than other biological parameters. Additionally, the distribution of Γ_{cdom} is closer to that of the physical parameters.

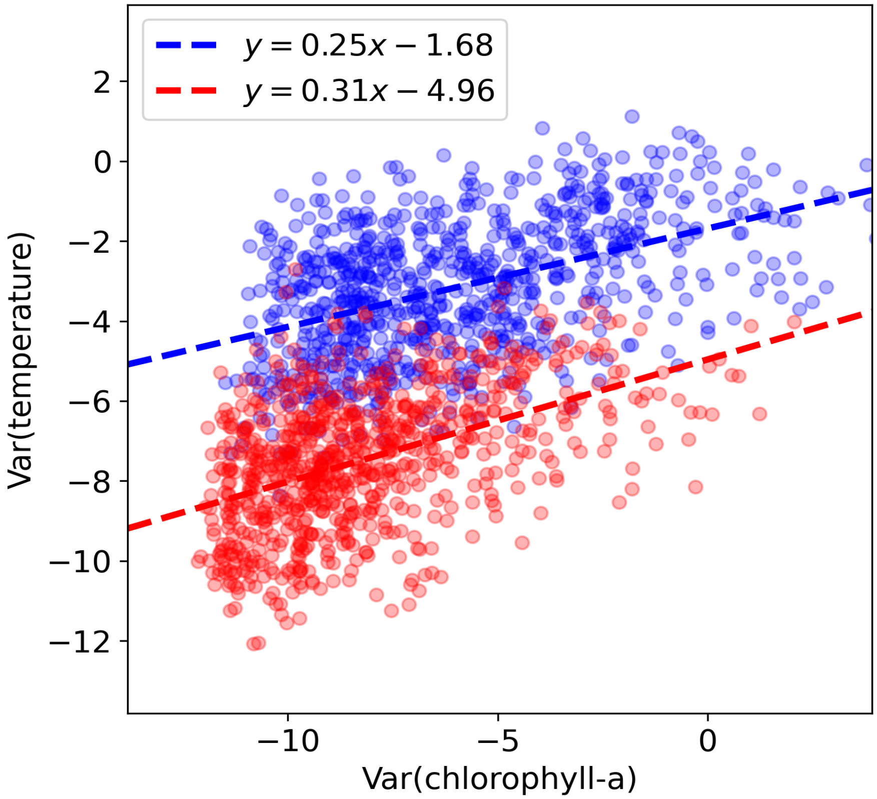

Figure S9. Temperature variance as a function of chlorophyll-a variance at the large scale (~75km, blue) and at the small scale (~0.6km, red). While the variances do have a correlation (R^2=0.22 in both cases), the variance slope (Γ) does not correlate between physical and biological variables (R^2=0.03).

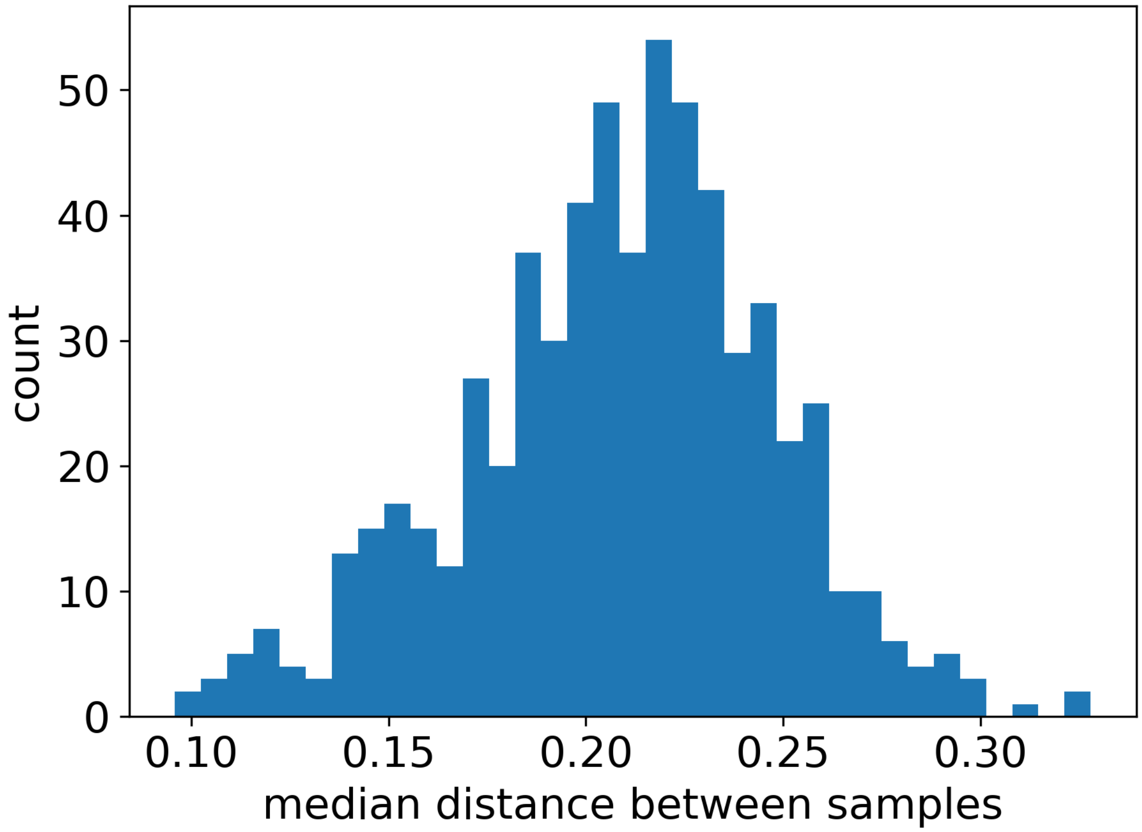

Figure S10. The median distance from one sample to the next for each transect. The median of these values is 0.21 km, leading to a median minimum analysis window of ~0.63km based on a minimum window size of 3 samples.
