# Supplementary material for: Emergent patterns of patchiness differ between physical and planktonic properties in the ocean
Source: Nat Commun. 2025 Feb 20;16:1808. doi: 10.1038/s41467-025-56794-x (PMC11842825; doi:10.1038/s41467-025-56794-x)
Supplement: Supplementary file 2 — Reporting Summary [file 41467_2025_56794_MOESM2_ESM.pdf]

nature portfolio

Corresponding author(s): *Double-anonymous peer review submissions: write DAPR and your manuscript number here instead of author names.*

Last updated by author(s): *YYYY-MM-DD*

Reporting Summary

Nature Portfolio wishes to improve the reproducibility of the work that we publish. This form provides structure for consistency and transparency in reporting. For further information on Nature Portfolio policies, see our Editorial Policies and the Editorial Policy Checklist.

Statistics

For all statistical analyses, confirm that the following items are present in the figure legend, table legend, main text, or Methods section.

n/a	Confirmed	
☐	☒	The exact sample size (*n*) for each experimental group/condition, given as a discrete number and unit of measurement
☐	☒	A statement on whether measurements were taken from distinct samples or whether the same sample was measured repeatedly
☐	☒	The statistical test(s) used AND whether they are one- or two-sided<br>*Only common tests should be described solely by name; describe more complex techniques in the Methods section.*
☐	☒	A description of all covariates tested
☐	☒	A description of any assumptions or corrections, such as tests of normality and adjustment for multiple comparisons
☐	☒	A full description of the statistical parameters including central tendency (e.g. means) or other basic estimates (e.g. regression coefficient) AND variation (e.g. standard deviation) or associated estimates of uncertainty (e.g. confidence intervals)
☐	☒	For null hypothesis testing, the test statistic (e.g. *F*, *t*, *r*) with confidence intervals, effect sizes, degrees of freedom and *P* value noted<br>*Give P values as exact values whenever suitable.*
☒	☐	For Bayesian analysis, information on the choice of priors and Markov chain Monte Carlo settings
☒	☐	For hierarchical and complex designs, identification of the appropriate level for tests and full reporting of outcomes
☐	☒	Estimates of effect sizes (e.g. Cohen's *d*, Pearson's *r*), indicating how they were calculated

Our web collection on statistics for biologists contains articles on many of the points above.

Software and code

Policy information about availability of computer code

Data collection	All data was collected using the open source python program inlinino: https://github.com/OceanOptics/Inlinino as detailed in the methods of the paper.
Data analysis	All analysis was done in python as detailed in the methods. All code necessary to generate the figures is on GitHub https://github.com/patrickcgray/spatial_patchiness_tara. To obtain a Docker image for running this in an identical environment to the one used in this study run “docker pull pangeo/pangeo-notebook:2024.04.05” from the command line with Docker Desktop running. A series of Jupyter Notebooks are provided in the above Github repo for an exact reproduction of all work in this study.

For manuscripts utilizing custom algorithms or software that are central to the research but not yet described in published literature, software must be made available to editors and reviewers. We strongly encourage code deposition in a community repository (e.g. GitHub). See the Nature Portfolio guidelines for submitting code & software for further information.

Data

Policy information about availability of data

All manuscripts must include a data availability statement. This statement should provide the following information, where applicable:

- Accession codes, unique identifiers, or web links for publicly available datasets
- A description of any restrictions on data availability
- For clinical datasets or third party data, please ensure that the statement adheres to our policy

All data is available in raw form at NASA’s SeaBASS archive https://seabass.gsfc.nasa.gov/search via the search keyword “Tara_Microbiome”. All data prepared and

formatted for this study is available on GitHub https://github.com/patrickcgray/spatial_patchiness_tara, easily ingestible as a geofeather at multiple stages of processing.

Research involving human participants, their data, or biological material

Policy information about studies with human participants or human data. See also policy information about sex, gender (identity/presentation), and sexual orientation and race, ethnicity and racism.

Reporting on sex and gender	*Use the terms sex (biological attribute) and gender (shaped by social and cultural circumstances) carefully in order to avoid confusing both terms. Indicate if findings apply to only one sex or gender; describe whether sex and gender were considered in study design; whether sex and/or gender was determined based on self-reporting or assigned and methods used.*<br>*Provide in the source data disaggregated sex and gender data, where this information has been collected, and if consent has been obtained for sharing of individual-level data; provide overall numbers in this Reporting Summary. Please state if this information has not been collected.*<br>*Report sex- and gender-based analyses where performed, justify reasons for lack of sex- and gender-based analysis.*
Reporting on race, ethnicity, or other socially relevant groupings	*Please specify the socially constructed or socially relevant categorization variable(s) used in your manuscript and explain why they were used. Please note that such variables should not be used as proxies for other socially constructed/relevant variables (for example, race or ethnicity should not be used as a proxy for socioeconomic status).*<br>*Provide clear definitions of the relevant terms used, how they were provided (by the participants/respondents, the researchers, or third parties), and the method(s) used to classify people into the different categories (e.g. self-report, census or administrative data, social media data, etc.)*<br>*Please provide details about how you controlled for confounding variables in your analyses.*
Population characteristics	*Describe the covariate-relevant population characteristics of the human research participants (e.g. age, genotypic information, past and current diagnosis and treatment categories). If you filled out the behavioural & social sciences study design questions and have nothing to add here, write "See above."*
Recruitment	*Describe how participants were recruited. Outline any potential self-selection bias or other biases that may be present and how these are likely to impact results.*
Ethics oversight	*Identify the organization(s) that approved the study protocol.*

Note that full information on the approval of the study protocol must also be provided in the manuscript.

Field-specific reporting

Please select the one below that is the best fit for your research. If you are not sure, read the appropriate sections before making your selection.

☐ Life sciences ☐ Behavioural & social sciences ☒ Ecological, evolutionary & environmental sciences

For a reference copy of the document with all sections, see nature.com/documents/nr-reporting-summary-flat.pdf

Ecological, evolutionary & environmental sciences study design

All studies must disclose on these points even when the disclosure is negative.

Study description	In this work we examine the spatial patterns, specifically the patchiness, of planktonic and physical parameters in the ocean. Planktonic variables are: chlorophyll-a (chla), particulate attenuation at 443nm (cp(443)), and mean particle size (γ). Physical variables are: temperature, density (σ), and salinity.<br><br>We quantify patchiness using variance slope which is similar to spectral analysis, but able to ingest vectors (i.e. transects) with small gaps within them. Variance slope is calculated on groups of 500 samples using a set of 11 log-distributed windows from 3 to 500. Variance and window size were log-transformed, and we ran a least-squares fit to find the slope of this line, the variance slope. We then analyze this parameter across space and compare it for the six variables above.
Research sample	The research sample is N=661,552 (dt=1 minute, dx~200m) measurements from the Atlantic, Pacific, and Southern Oceans meant to serve as a broad representation of the global ocean. This data is all from the inline system of the S/V Tara and was chosen because it is the largest available high quality, consistently collected dataset of its kind.
Sampling strategy	The sampling strategy was not a part of this research, but the data is extensive and covers nearly all possible geographic, temporal, and biogeochemical conditions in the ocean.
Data collection	All in situ measurements are from the S/V Tara underway sampling system which pulls seawater from 2m depth. Temperature and salinity data is from a Seabird Scientific thermosalinograph (SBE 38 and SBE 45) and all optical data is derived from particulate absorption and attenuation measured with a Seabird Scientific ACs. All data was processed by co-author Guillaume Bourdin.
Timing and spatial scale	The data was collected from 2016 to 2022. Data is collected at 4Hz and then processed to 1 minute averages for quality control and to remove samples contaminated with bubbles. Data is not collected when the ship is at port, undergoing maintenance, or when the

	system is being cleaned. Spatiotemporal scale is dt=1 minute, dx~200m.
Data exclusions	No data we excluded and the quality control process was done independently before the study was fully conceived. The quality control process is: For each minute of total seawater measurements (sampled at 4Hz) the signal between the 2.5th and 97.5th percentiles were averaged, and their standard deviation was used to quantify uncertainty. Dropping the 2.5th to 97.5th percentiles filters out noisy spikes from bubbles which can be a major problem in optical measurements. All spectra were manually checked and quality controlled for obviously bad measurements (e.g. bubbles, bad filtered seawater measurements).
Reproducibility	This is not really relevant, but we did compare our experimental results across the 820 transects that resulted after calculating the variance slope for the 661,552 minute binned measurements.
Randomization	Not relevant.
Blinding	Not relevant.

Did the study involve field work? ☒ Yes ☐ No

Field work, collection and transport

Field conditions	The conditions across Atlantic, Pacific, and Southern Oceans over six years varied widely but all instruments were in the same ship compartment and consistently collecting data from 2m depth.
Location	The location covers broad swaths of the Atlantic, Pacific, and Southern Oceans over six years.
Access & import/export	Not relevant.
Disturbance	Not relevant.

Reporting for specific materials, systems and methods

We require information from authors about some types of materials, experimental systems and methods used in many studies. Here, indicate whether each material, system or method listed is relevant to your study. If you are not sure if a list item applies to your research, read the appropriate section before selecting a response.

Materials & experimental systems

n/a	Involved in the study	
☒	☐	Antibodies
☒	☐	Eukaryotic cell lines
☒	☐	Palaeontology and archaeology
☒	☐	Animals and other organisms
☒	☐	Clinical data
☒	☐	Dual use research of concern
☒	☐	Plants

Methods

n/a	Involved in the study	
☒	☐	ChIP-seq
☒	☐	Flow cytometry
☒	☐	MRI-based neuroimaging

Plants

Seed stocks	*Report on the source of all seed stocks or other plant material used. If applicable, state the seed stock centre and catalogue number. If plant specimens were collected from the field, describe the collection location, date and sampling procedures.*
Novel plant genotypes	*Describe the methods by which all novel plant genotypes were produced. This includes those generated by transgenic approaches, gene editing, chemical/radiation-based mutagenesis and hybridization. For transgenic lines, describe the transformation method, the number of independent lines analyzed and the generation upon which experiments were performed. For gene-edited lines, describe the editor used, the endogenous sequence targeted for editing, the targeting guide RNA sequence (if applicable) and how the editor was applied.*
Authentication	*Describe any authentication procedures for each seed stock used or novel genotype generated. Describe any experiments used to assess the effect of a mutation and, where applicable, how potential secondary effects (e.g. second site T-DNA insertions, mosiacism, off-target gene editing) were examined.*
